# Molecular Characterization and Clinical Relevance of N^6^-Methyladenosine Regulators in Metastatic Prostate Cancer

**DOI:** 10.3389/fonc.2022.914692

**Published:** 2022-06-22

**Authors:** Qiwei Liu, Zhen Li, Lizhao He, Ke Li, Chen Hu, Jialiang Chen, Fangjian Zhou, Jun Wang, Yonghong Li, Hengjun Xiao

**Affiliations:** ^1^ Department of Urology, the Third Affiliated Hospital of Sun Yat-Sen University, Guangzhou, China; ^2^ Plastic Surgery Institute, Chinese Academy of Medical Sciences and Peking Union Medical College, Beijing, China; ^3^ State Key Laboratory of Oncology in South China, Collaborative Innovation Center for Cancer Medicine, Sun Yat-sen University Cancer Center, Guangzhou, China; ^4^ Department of Urology, Sun Yat-sen University Cancer Center, Guangzhou, China

**Keywords:** metastatic prostate cancer, m6A, regulator, prognosis, treatment

## Abstract

Prostate cancer is a leading malignancy in the male population globally. N6-methylation of adenosine (m6A) is the most prevalent mRNA modification and plays an essential role in various biological processes *in vivo*. However, the potential roles of m6A in metastatic prostate cancer are largely unknown. In this study, we evaluated and identified two m6A modification patterns based on 21 m6A regulators in four public metastatic prostate cancer datasets. Different modification patterns correlated with distinct molecular characteristics. According to m6A-associated genes, we constructed a prognostic model, called m6Ascore, to predict the outcomes of patients with metastatic prostate cancer. We found that high m6A score level was related to dismal prognosis and characterized by higher cell cycle, DNA repair and mismatch repair pathway score. *In vitro* experiments confirmed that upregulation of METTL14, an m6A writer, enhanced the invasion, metastasis, and sensitivity of prostate cancer cells to poly (ADP-ribose) polymerase inhibitor. Conversely, down-regulation of potential target genes of m6A had the opposite effect. Finally, we validated that a higher m6A score was associated with a worse prognosis and a higher Gleason score in The Cancer Genome Atlas Program (TCGA) cohort. This work illustrated the nonnegligible role of m6A modification in multiple biological processes of metastatic prostate cancer. Evaluating the m6A risk scores of individual tumours will guide more effective judgement of prognosis as well as treatments for metastatic prostate cancer in clinical practice.

## Introduction

Prostate cancer (PCa) is the most prevalently diagnosed malignancy in men. There are, however, limited effective treatments for advanced prostate cancer, especially metastatic prostate cancer (1). Although multiple treatments, including surgery, chemotherapy, radiotherapy, and targeted therapy, have improved the outcomes of prostate cancer to some extent, some adverse effects, such as resistance and toxicity, still exist (2). Thus, burrowing prognostic and therapeutic molecular biomarkers is urgent.

To date, more than 150 kinds of posttranscriptional modifications in RNA have been identified (3). N6-methyladenosine (m6A) is the most common RNA modification in mammalian cells (4) and has been suggested to be involved in various aspects of RNA metabolism and to play essential roles in different biological processes in mammals (5, 6). m6A methylation is achieved by recognition proteins (readers) and methyltransferases (writers), and the demethylation process is conducted by demethyltransferases (erasers). “Readers” include YTHDF1/2/3, YTHDC1/2, FMR1 and HNRNPA2B1; “writers” include METTL3, METTL5, METTL14, METTL16, WTAP, KIAA1429, ZC3H13 and RBM15; and “erasers” include FTO and ALKBH5.

Accumulated studies have highlighted tight connections between m6A methylation and tumour initiation and progression (6). In glioblastoma, downregulation of FTO or upregulation of METTL3 was involved in the poor prognosis of glioblastoma by promoting the proliferation and self-renewal of glioblastoma stem cells (7). High expression of METTL3 or METTL4 was also essential for the maintenance and self-renewal of leukaemia stem cells, thus aggravating acute myeloid leukaemia (8). Upregulation of METTL3 and downregulation of METTL14 can both lead to progression of hepatocellular carcinoma by facilitating cell proliferation and invasion (9, 10). YTHDF2 not only enhances cell proliferation by the AKT/GSK3β/cyclin D1 signalling axis but also inhibits migration and invasion by destabilizing the m6A sites of YAP (11). Huang et al. constructed a prognostic model for colon cancer basing on seven m6A regulators, and characterized three distinct subtypes of colon cancer, one of which was recognized as immunosuppressive (12). Similarly, Zhang et al. characterized tumor microenvironment characteristics through evaluating the m6A modification patterns (13). Wang et al. constructed a prognostic model for prostate cancer based on MRTTL14 and YTHDF2 (14). However, the mode of action of m6A methylation in metastatic prostate cancer remains largely unknown. Herein, we used published sequencing data to investigate the exact role of m6A methylation with respect to metastatic prostate cancer. This m6Ascore group-based model may facilitate the more effective judgement of prognosis for patients with metastatic prostate cancer and offer more valuable information for personalized precise pharmacy therapy.

## Materials and Methods

### Prostate Cancer Dataset

Public gene expression data and relative clinical information were gathered from the TCGA database (https://xenabrowser.net/datapages/). Patients without detailed survival information were removed. In addition, four eligible metastatic prostate cancer cohorts were acquired from https://www.cbioportal.org/, which include mRNA expression data, somatic mutation data and copy number variation (CNV). Clinical annotations were downloaded by the *R* package cgdsr, and somatic mutation data were collected using the R package TCGAbiolinks (15). Specific collected data are shown in **Table 1**, and more detailed information about the samples is presented in **Supplementary Table 1**.

**Table 1:** Specimen information.

	mRNA	SNP	CNV
TCGA_PRAD	481	503	502
nepc_wcm_2016	49	114	107
prad_mich	31	61	60
prad_su2c_2015	118	150	150
prad_su2c_2019	212	442	443

For data consistency, the original data from https://www.cbioportal.org/were normalized by the z-score function, and the FPKM data from TCGA were transformed into the zscore normalized dataset. Finally, batch effects were corrected using the *R* package *sva*.

### Unsupervised Clustering for 21 m6A Regulators

Altogether, 21 m6A regulators were extracted from four eligible metastatic prostate cancer cohorts downloaded from the cBioPortal website to discern distinct m6A regulator-mediated modification patterns. These regulators consisted of 8 writers (METTL3, METTL14, RBM15, RBM15B, WTAP, KIAA1429, CBLL1, ZC3H13), 2 erasers (ALKBH5, FTO) and 11 readers (YTHDC1, YTHDC2, YTHDF1, YTHDF2, YTHDF3, IGF2BP1, HNRNPA2B1, HNRNPC, FMR1, LRPPRC, ELAVL1). Based on the different expression patterns of m6A regulators, unsupervised clustering was performed to identify various m6A modification patterns and classify patients. We applied the consensus clustering algorithm (ConsensuClusterPlus package, 1000 repetitions) to determine cluster numbers and their stability (16).

### Gene Set Variation Analysis and Functional Annotation

To further investigate the biological significance of different m6A modification patterns, we conducted GSVA enrichment analysis with the “GSVA” R package. GSVA is a nonparametric and unsupervised technique that is commonly used to estimate changes in biological processes and signal pathways in samples (17). The annotated gene sets of “c2.cp.kegg.v6.2.-symbols” were collected from the MSigDB database (https://www.gsea-msigdb.org/gsea/index.jsp). Adjusted P <0.5 was viewed as statistically significant. To carry out functional annotation for m6A-related genes, the clusterProfiler R package was used (FDR cut-off of < 0.05).

### Identification of Differentially Expressed Genes Between Distinct m6A Phenotypes

Referring to distinctly expressed m6A regulators, we classified four eligible metastatic prostate cancer cohorts collected from the cBioPortal website into two different m6A modification patterns. DEGs between the two distinct modification patterns were determined by the R package limma (18). Genes with p<0.5 and 1.5<fold-change (or fold-change <0.667) were regarded as differentially expressed genes.

### m6Ascore Calculation

Redundant genes of DEGs were removed using the random forest approach (19), and the remaining genes were selected for survival analysis (p<0.05). Genes were classified into two clusters utilizing the Cox regression model. Based on the above genes, we construct a prognostic model, called m6Ascore.We then calculated m6Ascore referring to the following GGI method (20): m6Ascore=scale(∑X-∑Y), where x or y is the gene expression value when the Cox coefficient is positive or negative, respectively. Based on the median value of m6Ascore, samples were divided into m6Ascore-high and m6Ascore-low. Subsequently, prognostic analysis was performed between the two samples.

### Correlation Between the m6A Gene Signature and Other Related Biological Processes

Mariathasan et al. constructed a series of gene sets involved in specific biological processes, including immune checkpoints; epithelial mesenchymal transition (EMT) markers such as EMT1 and EMT2; and DNA mismatch repair (21). We subsequently carried out correction analysis to uncover the relationships between m6Ascore and relative biological pathways.

### Copy Number Variation Analysis

According to SNP6 CopyNumber segment data, the shared changing areas of copy number among all the samples were detected utilizing the GISTIC method. Relative parameters were set as follows: Q ≤ 0.05, confidence level was 0.95. The above analysis was performed using the corresponding MutSigCV module of GenePattern (https://cloud.genepattern.org/gp/pages/index.jsf, an online analytical tool developed by the Broad Research Institute.

### Cell Culture and Cell Transfection

Human prostate cancer cell lines DU145 and PC3 were obtained from ATCC (USA). Cells were kept in RPMI-1640 medium supplemented with 10% FBS at 37°C in a humidified incubator with 5% CO_2_.

After reaching 80% confluency, cells were transfected with the following lentiviral plasmids using Lipofectamine^®^ 2000 (Invitrogen): short hairpin (sh)RNA-NC (5 nM), pLVSO2-METTL14 (5 nM), pLKOG-shRNA-CSNK1D-ABC (5 nM), pLKOG-shRNA- METTL14-AB (5 nM), and pLKOG-shRNA-SLC35E1-ABC (5 nM). Twenty-four hours after transfection, subsequent experiments were performed.

### Western Blot Analysis

Western blotting was conducted as previously described (22). Briefly, protein concentrations were measured with a BCA Kit. Protein lysates were resolved using SDS–PAGE and transferred onto PVDF membranes (Millipore). The membrane was subsequently incubated overnight (4°C) with the following primary antibodies: anti-METTL14 (Norvus), anti-CSNK1D (Norvus), anti-SLC35E1 (Norvus) and β-actin (Invitrogen). After washing, the membranes were further subjected to the appropriate secondary antibodies (Invitrogen). Blots were visualized by a ChemiDoc XRS system, followed by quantification using Image Lab software (Bio–Rad).

### Transwell Assay

Matrigel was defrosted at 4°C overnight and diluted with serum-free medium (dilution, 1:6). Transwells were inserted in a 24-well culture plate, 40 µl of prediluted Matrigel was inoculated into each Transwell chamber, followed by 2 hours in a 37°C incubator to coagulate. Stably transfected cells were previously seeded in 6-well plates and cultured to 90% confluence. After digestion, a total of 200 µl cell suspension (8×10^4^ cells/well) was dispensed to the upper chamber, and 800 µl medium containing 30% FBS was dispensed to the lower chamber. After 24 hours of incubation at 37°C, cells in the upper layer of the Transwell were removed with sterile cotton swabs, followed by PBS washing and fixation with methanol for 20 min. Subsequently, cells were further stained with crystal violet dye for 5 min, washed with distilled water, imaged and counted under an inverted microscope.

### Wound Healing Assay

Transfected cells were plated into a 6-well plate. Before scratching, the culture medium was replaced with serum-free medium containing 1 μg/ml mitomycin C to obtain monolayer cells. Scratches were generated using 200 µl pipette tips, followed by washing three times with PBS. Migrated cells were counted and photographed by a microscope at 0 and 24 hours after scratching.

### CCk-8 Assay

When the cell confluency reached 70%, drugs were added for 72 hours. DMSO was added to the control groups, and the experimental groups were administered olaparib for 72 hours. Cells were cultured to 90% confluence and then subjected to digestion, centrifugation and resuspension. Cells were further seeded in 96-well plates at a density of 4×103 cells/well. Cell proliferation was detected with a CCK-8 assay following the manufacturer instructions after culture for 24, 48 and 96 hours. The absorbance was measured at 45 nm wavelength.

### Statistical Analyses

The bioinformatics differences between the two groups were analysed using the Wilcox test. Referring to the relevance between m6Ascore and patient survival, the cut-off values of different subgroups were identified by the survminer R package. Survival curves were generated using Kaplan–Meier analysis, and significant differences were determined by log-rank tests. The predictive value of m6Ascore for metastatic samples was evaluated *via* receiver operating characteristic (ROC) curve analysis, and the area under the curve (AUC) was calculated utilizing the pROC R package. The maftools R package was applied to plot the mutation atlas of patients with high and low m6Ascore. The R package RCircos was used to depict the location of m6A regulators on chromosomes. ns represents P > 0.05, *P ≤0.05, ** P ≤ 0.01, *** P ≤ 0.001, **** P ≤0.0001.

For the experimental data, a two-tailed t test was used with PRISM software. A P value < 0.05 was viewed as statistically significant.

## Results

### The Genetic Variation of m6A Regulators

**Figure 1:**
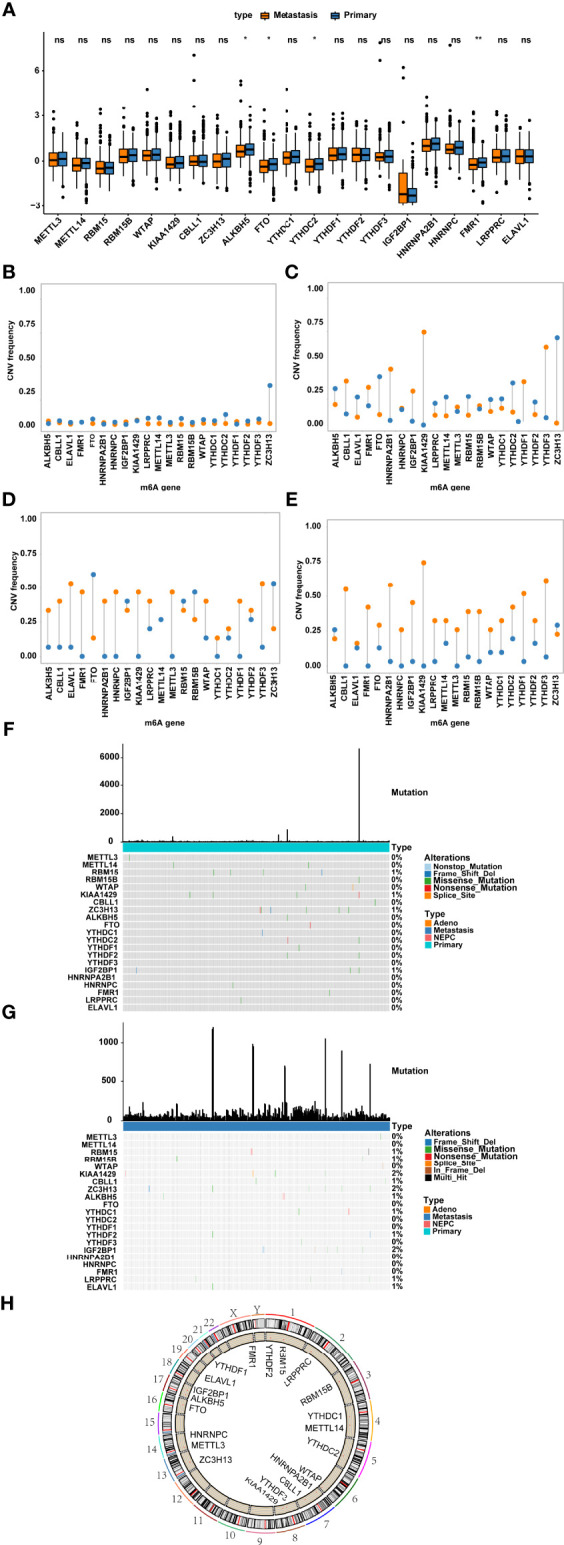
Genetic variants of m6A regulators. **(A)** The expression of m6A regulator genes in nonmetastatic and metastatic prostate cancers; Frequency of CNV in m6A regulator genes in primary tumour **(B)**, metastatic tumour **(C)**, neuroendocrine prostate cancer **(D)** and prostate adenoma **(E)** were shown. Blue represents deletion, orange represents amplification. **(F, G)** The location of somatic mutations of m6A regulator genes in **(F)** primary tumour and **(G)** metastatic tumour. **(H)** The location of m6A regulator genes on chromosomes. ns represents P > 0.05, *P ≤ 0.05, **P ≤ 0.01.

Altogether, 21 m6A regulators (8 writers, 2 erasers and 11 readers) were identified. We first analysed the mRNA expression levels of m6A regulators between metastatic and nonmetastatic samples and found that few genes were differentially expressed, such as FMR1 and FTO (**Figure 1**). Subsequently, we summarized the incidence of CNV and somatic mutations of 21 m6A regulators in metastatic, nonmetastatic and NEPC samples. Except for the prevalent missing frequency of CNV in a few regulators, such as FTO, RBM15B and YTHDC2, most regulators experienced an amplification in copy number (**Figures 1B–E**; **Supplementary Table 2**). Among these samples, mutations of m6A regulators rarely occurred (**Figures 1F, G**). The distribution of m6A regulators on chromosomes is presented in **Figure 1H**.

### Unsupervised Clustering for m6A Regulators in Metastatic Prostate Cancer

**Figure 2:**
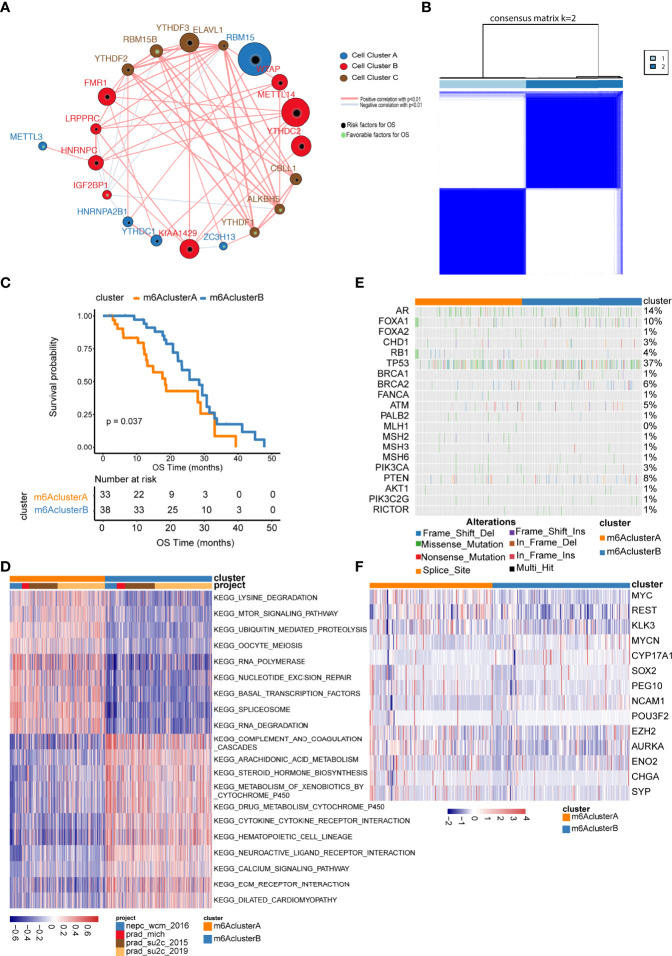
Unsupervised clustering of m6A regulator genes in metastatic prostate cancer. **(A)** The interaction among m6A regulator genes. The size of circle indicates the effect of each gene on survival, the larger the size, the greater the effect is; green spots inside the circle indicate risk prognostic factors, black spots inside the circle indicate factors; lines that connect genes exhibit genetic interactions, red and blue represent positive and negative associations, respectively; gene Cluster A, B and C are shown as blue, red and brown, respectively; **(B)** Consensus clustering m6A regulator genes in metastatic samples; **(C)** Kaplan–Meier curves indicate that there is a strong relationship between the m6Acluster types and the overall survival rate; **(D)** GSVA enrichment analysis. Heatmaps show the activation status of biological pathways, which is displayed with different m6A clusters; red denotes activation, blue denotes inhibition; **(E, F)** show the distribution of the mutation and expression of partial genes in two m6A clusters, respectively.

We performed consensus clustering and univariate Cox analysis utilizing m6A gene expression matrix and patient’s survival information from the prad_su2c_2019 dataset. The m6A regulation network in **Figure 2A** (**Supplementary Table 3**) revealed that the interaction and junction of m6A regulators and their impacts on the prognosis of metastatic prostate cancer. We found that not only the same functional categories of m6A regulators but also the distinct functional categories of m6A regulators displayed significant correlations in expression.

The above results illustrated that the interactions between distinct functional categories of m6A regulators may play important roles in various m6A modification patterns. We characterized the different expression patterns of 21 m6A regulators in four eligible metastatic prostate cancer cohorts downloaded from the cBioPortal website and performed unsupervised clustering analysis using the ConsensusClusterPlus R package, which led to the identification of two distinct subclusters (**Figure 2B**, **Supplementary Table 4**). We termed these patterns m6A Clusters.A and m6A Clusters.B, respectively.

To investigate biological behaviours among different subgroups, we performed gene set enrichment analysis (GSEA) (**Supplementary Table 5**). As shown in **Figure 2D**, m6A Cluster.A was significantly enriched in lysine degradation and the mTOR signalling pathway. m6A Cluster.B was mainly enriched in arachidonic acid metabolism and steroid hormone biosynthesis (**Supplementary Table 6**).

**Figure 3:**
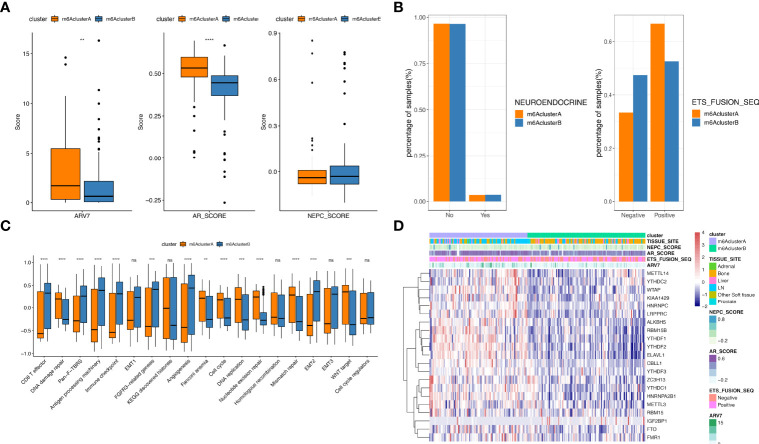
Comparison analysis among m6A clusters. **(A)** The distribution of ARV7 (left), ARscore (middle) and NEPCscore (right) between the two m6A clusters; **(B)** The prognostic differences between the two m6A clusters; **(C)** Results for GSVA analysis of prad_su2c_2019 cohorts; **(D)** The expression of m6A regulator genes in two m6A clusters extracted from prad_su2c_2019 cohorts. ns represents P > 0.05, **P ≤ 0.01, ***P ≤ 0.001, ****P ≤ 0.0001.

Furthermore, we evaluated the expression and mutation atlas of specific genes between m6A Cluster.A and m6A Cluster.B (**Figure 2E, F**, **Supplementary Tables 7, 8**). Particularly, in the prad_su2c_2019 datasets, the ARV7 score and ARscore between these two clusters showed significant differences (**Figure 3**, **Supplementary Table 9**). Further prognosis analysis revealed that the prognosis between these two clusters was significantly different (**Figure 2C**). Subsequently, we performed GSVA based on the gene sets constructed by Mariathasan et al. (**Figure 3C**, **Supplementary Table 10**). The activities of matrix molecules were markedly increased in m6A Cluster.B, such as the activation of epithelial mesenchymal transition, transforming growth factor-β and angiogenesis signalling pathways. In addition, the expression levels of m6A regulators in the m6A cluster.A were higher than in m6A Cluster.B (**Figure 3D**, **Supplementary Table 11**).

### m6A Regulators Promote PCa Cell Metastasis and Proliferation

**Figure 4:**
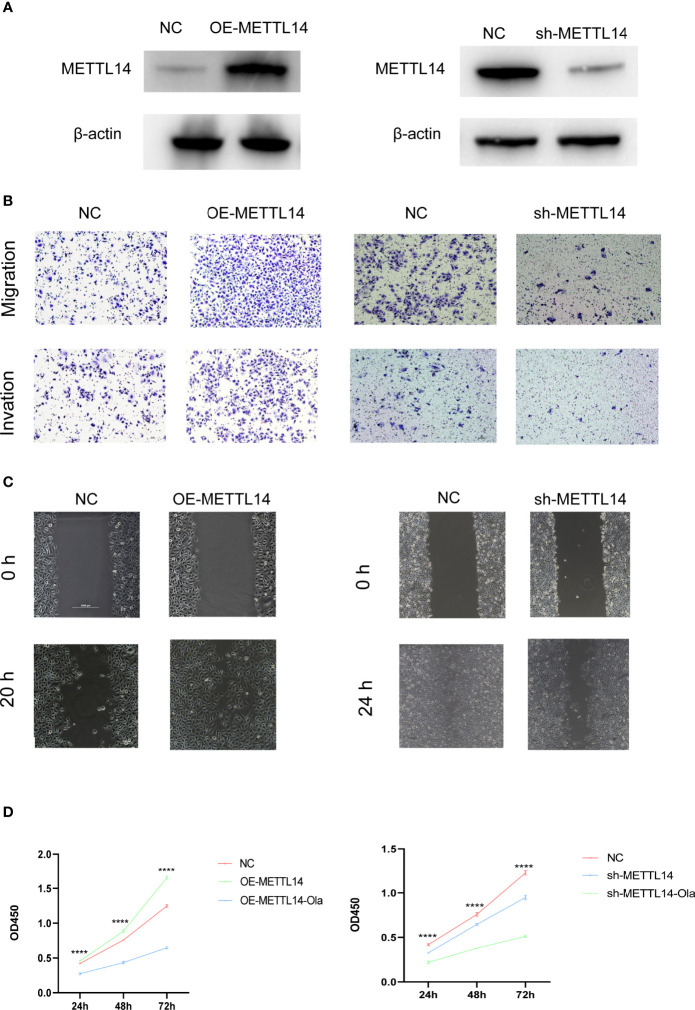
METTL14 promotes PC3 cell metastasis and proliferation *in vitro*. **(A)** Western blot analysis of METTL14 expression levels in METTL14-downregulated, METTL14-knockdown, and vehicle control cells. **(B)** Representative images of migration (upper panels) and invasion (lower panels) assays using PC3 cells, presenting cell migration and invasion after overexpression or knockdown of METTL14. **(C)** Wound healing assays using PC3 cells presenting cell motility after overexpression or knockdown of METTL14. **(D)** Cell proliferation was evaluated in METTL14-overexpressing (left) or METTL14-knockdown (right) PC3 cells with or without olaparib administration. ****P ≤ 0.0001.

To further investigate the function of m6A regulators during the metastasis of PCa, METTL14-overexpressing or METTL14 knockdown PC3 cell lines were established by transfecting a stable overexpressing lentivirus and shRNA, respectively. The efficiency of METTL14 knockdown and overexpression was validated by western blotting. The results revealed that the protein levels of METTL14 were significantly increased or decreased in PC3 cell lines (**Figure 4A**). Subsequently, cell migration, invasion, wound healing, and CCK-8 assays were performed to explore the role of METTL14 in PCa cell metastasis and proliferation, respectively. Cell migration and invasion assays showed that downregulation of METTL14 decreased the migration and invasion cell numbers, while overexpressing METTL14 reversed the outcomes (**Figure 4B**). Wound healing assays revealed that silencing METTL14 reduced, whereas overexpressing METTL14 increased, the wound healing of PC3 cells (**Figure 4C**). Moreover, the proliferation of PC3 cells was detected by CCK-8 assay, which elucidated that METTL14 ablation inhibited, while upregulating METTL14 enhanced the proliferation capability of PCa cells (**Figure 4D**). Additionally, olaparib administration obviously reversed the cell proliferation promoted by METTL14 overexpression. Overall, our results implied that METTL14 played an essential role in PCa migration, invasion and proliferation.

### Generation of m6A Phenotype Genes and Function

To further investigate each m6A cluster’s potential biological behaviours, we characterized 2330 metastatic prostate cancer-related differentially expressed genes (DEGs) using the limma package (**Supplementary Table 12**). The clusterProfilter package was utilized to perform KEGG analysis for DEGs, which indicated the enrichment of shearing and RNA transportation (**Supplementary Table 13**). Then, basing on the 2330 m6A phenotype-related DEGs, unsupervised clustering analysis was performed to classify patients with metastatic prostate cancer, which could be similarly divided into two subtypes termed the m6AGenecluster.A and m6AGenecluster.B (**Supplementary Table 14**). We observed that the expression levels of most m6A regulators were higher in m6AGenecluster.A than in m6AGenecluster.B and the prognosis of m6AGenecluster.A type tumours was poorer than of those of m6AGenecluster.B (**Figure S1C**).

### Establishment of the Prognostic Model

**Figure 5:**
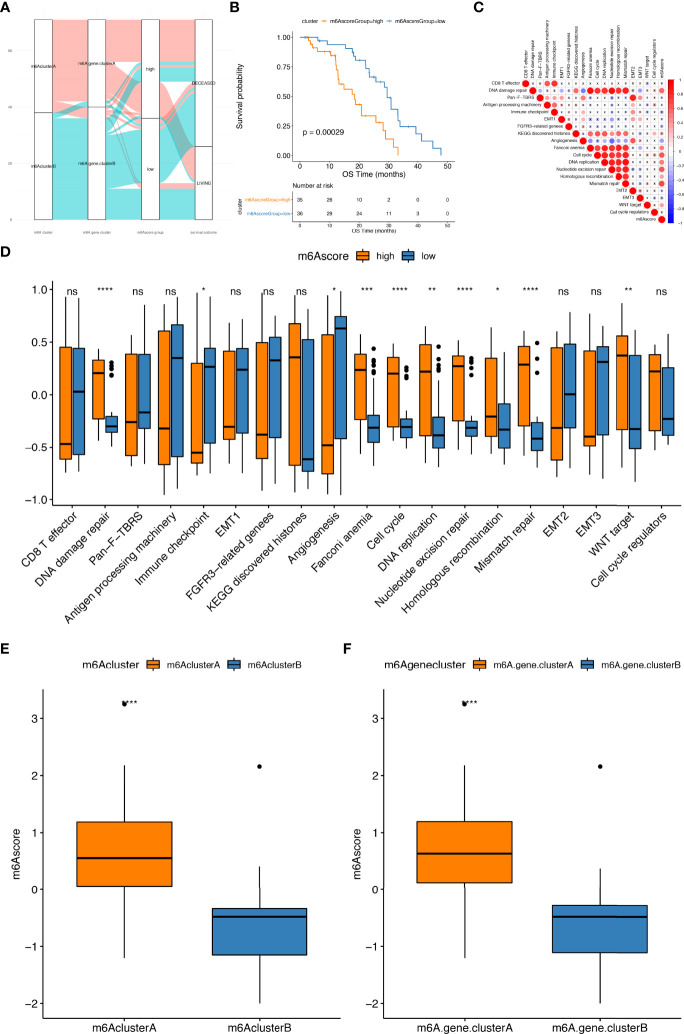
Construction of m6A risk score model. **(A)** The alluvial plot shows the changes of m6A clusters, gene clusters and m6Ascore; **(B)** Kaplan–Meier curves indicate that there is a strong relationship between the m6Ascore and the overall survival rate; **(C)** Pearson’s correlation analysis highlighting the correlations between m6Ascore and the known gene ontologies in prad_su2c_2019 cohorts. Red, blue and X symbols represent positive, negative and nonsignificant, respectively; the larger the circle, the more significant there is; **(D)** The distribution of enrichment scores of known gene ontologies prad_su2c_2019 cohorts between high and low m6Ascore samples; **(E, F)** show the distribution of m6Ascore among m6Aclusters and m6Ageneclusters, respectively. ns represents P > 0.05, *P ≤ 0.05, **P ≤ 0.01, ***P ≤ 0.001, ****P ≤ 0.0001.

The above DEGs were made de-redundant by the random forest algorithm to select the most category-related genes (**Supplementary Table 15_sig.txt**). The Cox regression model was used to uncover the relationship between these genes and patient’s survival. Next, we divided the above genes into two categories based on their coefficient values and scored for all the samples using the m6Ascore formula (**Supplementary Table 15_nGenes.txt; Supplementary Table 15_pGenes.txt**). Referring to the median m6Ascore, samples were further grouped into two categories: m6Ascore high and m6Ascore low samples (**Figure 5A**; **Supplementary Tables 16, 17**). As shown in **Figure 5B**, the prognosis of the m6Ascore high sample group was poorer than that the m6Ascore low group. This means that the prognosis of samples could be characterized by our m6Ascore model. Finally, the correlation analysis of m6Ascore and feature genes selected from gene sets constructed by Mariathasan et al. revealed that the m6Ascore was significantly associated with biological functions such as DNA repair and mismatch repair which imply the potetainal response to poly (ADP-ribose) polymerase (PARP) inhibitors (PARPis) (**Figure 5C, D**; **Supplementary Table 18**). The Wilcoxon test indicated that there was a notable difference between m6A cluster and m6AGene cluster in m6Ascore (**Figures 5E, F**). m6A risk scores of samples with enrichment of m6A cluster.A genes or m6AGene cluster.A genes were markedly higher than those of samples with highly expression of m6Acluster.B genes or m6AGenecluster.B genes.

**Figure 6:**
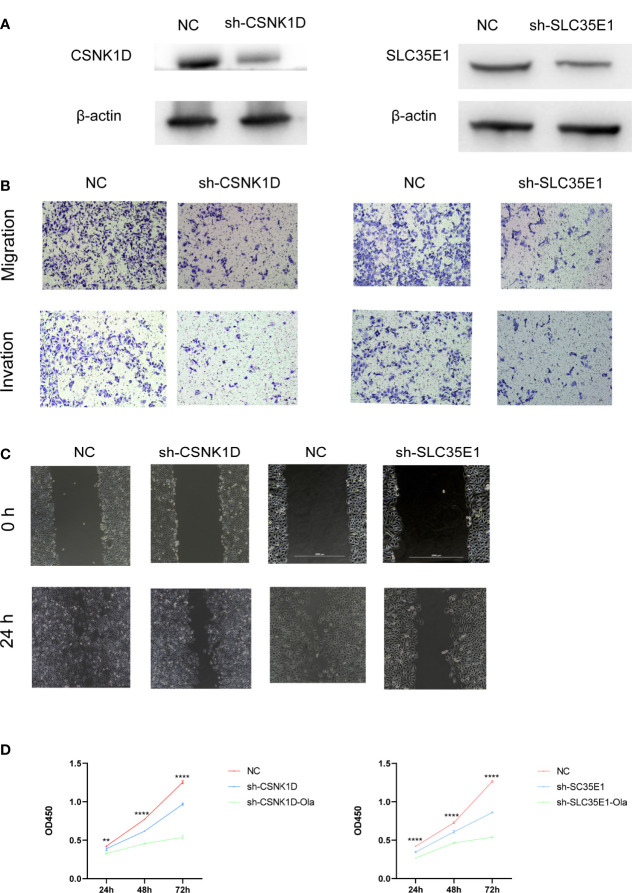
CSNK1D or SLC35E1 ablation promotes PC3 cell metastasis and proliferation *in vitro*. **(A)** Western blot analysis of CSNK1D or SLC35E1 expression levels in CSNK1D or SLC35E1 knockdown cells and vehicle control cells. **(B)** Representative images of migration (upper panels) and invasion (lower panels) assays using PC3 cells, presenting cell migration and invasion after knockdown of CSNK1D or SLC35E1. **(C)** Wound healing assays using PC3 cells presenting cell motility after knockdown of CSNK1D or SLC35E1 ablation. **(D)** Cell proliferation was evaluated in CSNK1D (left) or SLC35E1 (right) ablated PC3 cells with or without olaparib administration. **P ≤ 0.01, ****P ≤ 0.0001.

Similarly, we investigated the function of category-related genes during the metastasis of PCa. PC3 cell lines were stably transfected with lentiviruses expressing control shRNA, CSNK1D shRNA and SLC35E1 shRNA. The efficiency of CSNK1D or SLC35E1 knockdown was verified by western blotting (**Figure 6A**). Then, cell migration, invasion, wound healing, and CCK-8 assays were performed to explore the role of CSNK1D and SLC35E1 in PCa cell metastasis and proliferation, respectively. Cell migration and invasion assays showed that downregulation of CSNK1D and SLC35E1 decreased the migration and invasion cell numbers (**Figure 6B**). Wound healing assays also revealed that silencing CSNK1D or SLC35E1 reduced the wound healing abilities of PC3 cells (**Figure 6C**). Moreover, proliferation of PC3 cells was evaluated by CCK8 assay, which showed that CSNK1D or SLC35E1 ablation inhibited the proliferative capability, and administration of olaparib further inhibited the proliferative of PCa cells in the CSNK1D or SLC35E1 ablation groups (**Figure 6D**). In summary, our results revealed that both CSNK1D and SLC35E1 were of great significance in PCa migration, invasion, and proliferation.

### Molecular Characteristics Between High and Low m6Ascore

**Figure 7:**
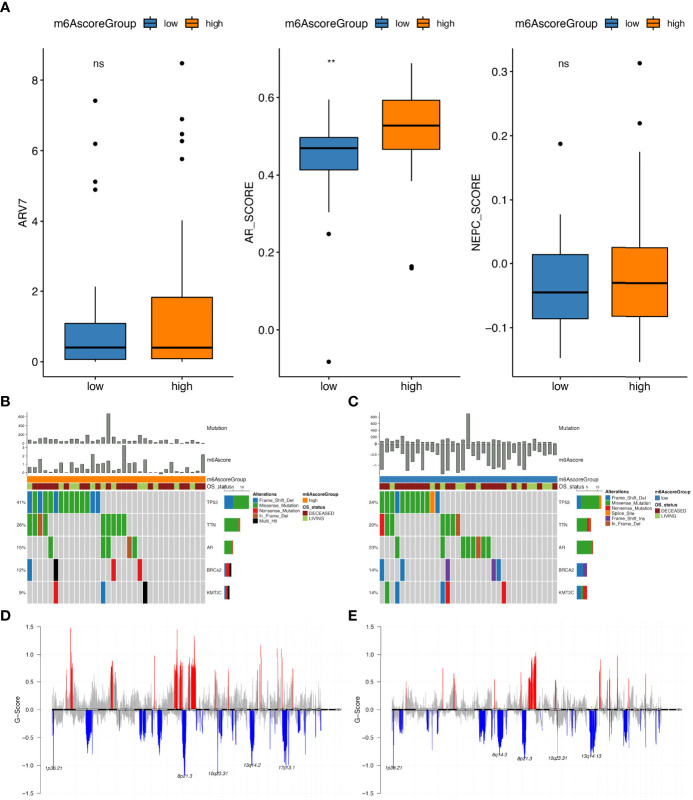
Molecular profiling of sample groups with high and low m6Ascore. **(A)** The distribution of ARV7 (middle), ARscore (middle) and NEPCscore (right) between samples with high and low m6Ascore; Gene mutation distribution of high **(B)** and low **(C)** m6Ascore samples; The distribution of copy number amplifications and deletions in high **(D)** and low **(E)** m6Ascore samples. ns, no significance; **P ≤ 0.01.

Additional investigations of differences between high and low m6Ascore groups in prad_su2c_2019 datasets revealed that the ARscore in different groups were distinct; in the high m6Ascore group, the ARscore was also high (**Figure 7A**). Then, we analysed the difference in somatic mutations between groups with high and low m6Ascore. As depicted in **Figures 7B, C**, the mutation numbers in the high m6Ascore groups were higher than those in the low m6Ascore group. Similarly, CNV numbers were also higher in the high m6Ascore groups than in the low m6Ascore groups (**Supplementary Table 19**). In the m6Ascore high groups, 18 copy number amplifications and 31 copy number deletions were detected, while in the low m6Ascore groups, 16 copy number amplifications and 30 copy number deletions were detected (**Figures 7D, E**).

### Verification of m6Ascore

**Figure 8:**
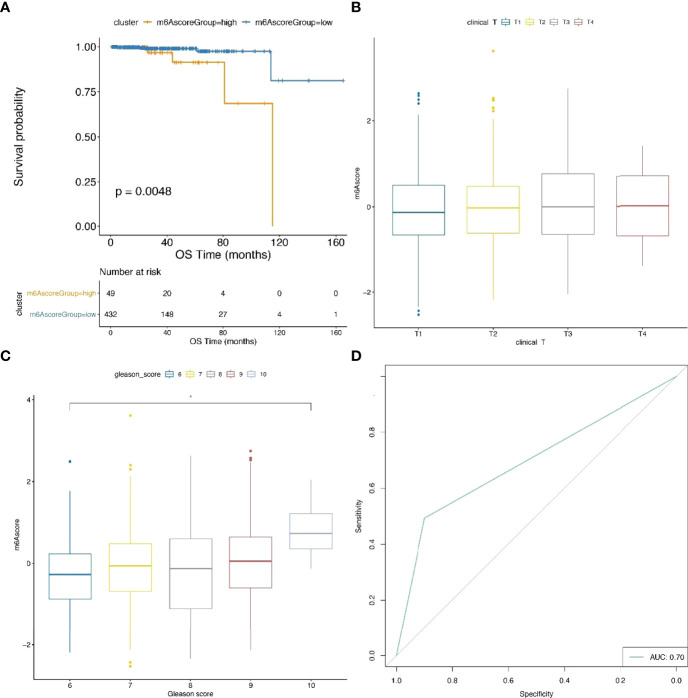
Comparison analysis and validation of m6Ascore model. **(A)** Survival analysis plot indicates a significant difference between TCGA samples with high and low m6A score. **(B, C)** The distribution of m6A score within distinct T stages and GLEASON_SCORE subgroups using TCGA data. **(D)** ROC curves for the prediction of metastatic and nonmetastatic prostate cancer between groups with high and low m6A score.

To further validate the predictive performance of our prognostic model, the m6A risk scores of TCGA samples were calculated. The threshold of classification was determined by the R function surv_cutpoint. Consistently, survival analysis indicated that the prognosis of the m6Ascore-high group was poorer than the m6Ascore-low group (**Figure 8A**; **Supplementary Table 20**). Furthermore, the m6Ascore also showed a significant difference in parts of the GLEASON_SCORE groups (**Figures 8B, C**).

In particular, we trained our prostate cancer metastasis prediction model in the prad_su2c_2019 and TCGA cohorts, which achieved an ROC AUC of 70% (**Figure 8D**). This indicated that our m6A risk score is efficient for the prognosis of metastatic prostate cancer.

## Discussion

PCa is a major malignancy affecting the male population worldwide, and effective therapeutic options for advanced-stage PCa, especially metastatic PCa, are still scarce (23). As the most wide-ranging posttranscriptional modification, m6A is strongly correlated with cancer cell proliferation, progression and metastasis (6). In PCa, however, relevant studies are still lacking, and there are no effective prediction models based on m6A regulators to evaluate the prognosis of metastatic PCa.

In our study, we found that the mRNA expression of most genes did not exhibit prominent differences between primary and metastatic samples, except for a few genes such as FMR1 and FTO. We also performed integrative analysis on primary, metastatic and NEPC prostate cancer samples basing on the CNVs and mutation alterations and mRNA expression of m6A regulators. Although few mutations were observed, their biological significance had been verified to be vital during tumour progression. A mutation in METTL14 could facilitate tumour proliferation *via* the AKT signalling pathway (24). There is a paucity of studies focusing on mutations of m6A regulators in PCa, but in acute myeloid leukaemia, mutations of m6A regulators were predictive of unfavourable prognosis (25). CNVs are strongly related to mRNA expression. Specifically, copy number gain could foster amplification of genes, and copy number reduction inhibits the expression of genes. Except for a few regulators, such as YTHDF2, FTO and RBM15B, most of them experienced CNV amplification. Amplification of FTO was reported to significantly improve the prognosis of prostate cancer (26).

However, the same m6A regulator may exert different roles in distinct tumours through diverse mechanisms. Herein, two distinct molecular subgroups of metastatic prostate cancer with obviously distinct characteristics were shown based on 21 m6A regulators related to prognosis. m6Acluster.A regulators were significantly enriched in lysine degradation and the mTOR signalling pathway. While the m6Acluster.B regulators were mainly enriched in arachidonic acid metabolism and steroid hormone biosynthesis. It’s well known that activating mTOR signalling can enhance tumour proliferation and progression *via* distinct mechanisms, including the enhancement of angiogenesis, glyolytic and lipid metabolism, and inhibition of autophagy (27). Additionally, the expression level of m6A regulator was higher in m6A clusters. A than in m6Acluster.B. To further investigate the relationship between the expression of m6A regulators and PCa prognosis, METTL14-overexpressing or METTL14 knockdown PC3 and DU145 cell lines were constructed. Similar to previous studies, METTL14 ablation inhibited the proliferation and metastasis capability, while upregulating METTL14 enhanced the proliferation and metastasis of PCa cells (28).

Furthermore, in our study, the transcriptomic heterogeneity among distinct subgroups of metastatic prostate cancer was found to be markedly related to shearing and RNA transportation. A total of 2330 DEGs were presented as m6A phenotype-related genes. Similar to m6A regulator clustering results, two distinct genomic subtypes were identified based on m6A phenotype-related genes (2330). Prognosis in m6AGenecluster.A type tumour was dismal, and the expression level of most regulators in the m6A cluster. A were higher than m6AGenecluster.B. Next, we selected the most category-related genes based on the above DEGs and then constructed a prognostic model to provide a reference for treating patients with metastatic prostate cancer. We observed that the m6Ascore was significantly correlated with some biological functions such as DNA repair and mismatch repair. Similarly, the m6A risk scores of samples with upregulated m6Acluster.A regulators or m6Agenecluster.A genes were distinctively higher than samples overexpressing m6Acluster.B regulators or m6Agenecluster.B genes. This work implied that m6A regulators play an essential role in the prognosis of metastatic PCa, and patients with high m6A risk scores may be more appropriate for targeted therapy against DNA repair mechanisms such as PARPi.

Androgen receptor (AR) plays an important role in the occurrence and development of prostate cancer, and when it is activated by androgen, it can regulate the expression of downstream target genes, thus promoting the progression and metastasis of prostate cancer. As our results showed, in the high m6Ascore groups, the ARscore, mutation and CNV numbers, which were unfavourable factors for prognosis, were correspondingly elevated. In this model, CSNK1D is located on chromosome 17. Gene expression and activity changes of CSNK1D have been observed in distinct cancers (29). In metastatic HCC, the expression level of CSNK1D was higher than that in nonmetastatic HCC (30). SLC35E1 (solute carrier family 35, member E1) is a nucleotide sugar transporter carrier. It has been reported that during colorectal liver metastasis, SLC35E1 could be a predictive factor for the therapeutic effect of 5-fluorouracil–based chemotherapy (31). In our validation experiment, silencing CSNK1D or SLC35E1 reduced the proliferation and metastasis of DU145 and PC3 cells, which showed similar effects to the vehicle groups that were administered olaparib. Furthermore, KDM1A, the first identified demethylase, also termed LSD1 or KIAA0601, can regulate the initiation of tumours (32). CCCTC-binding factor (CTCF) is a well-known regulator facilitating chromatin into topologically associated domains by enhancing cohesin-mediated loop formation (33), which is strongly associated with cancer initiation (34). RBBP4 could promote the malignant progression of colon cancer through the Wnt/β-catenin pathway (35). CDC23 regulates the tumour cell phenotype and is upregulated in papillary thyroid cancer (36). Cell division cycle 5-like (CDC5L) protein, a cell phase regulator of the G2/M transition, has been demonstrated to improve bladder cancer cell proliferation, migration and invasion (37). As an RNA-binding protein, hnRNPA1 can regulate the expression and translation of several mediators involved in tumour initiation and progression (38).

In this model, m6A risk score was positively correlated with Gleason score, an index widely used for the prognosis of prostate cancer, and negatively correlated with the survival time of patients with metastatic prostate cancer. These signified that our prognostic model is effective for the prognosis of metastatic prostate cancer. However, there was no significant association between the m6A risk score and T stages. In short, the prognostic model could be applied to guide more effective judgement of prognosis as well as treatment effects of metastatic prostate cancer in clinical practice. For metastatic PCa patients, a high m6A risk score indicates a dismal prognosis. Since the m6Ascore was significantly correlated with biological functions such as DNA repair and mismatch repair, patients with high m6Ascores may be appropriate candidates for pharmacy therapy targeted for DNA repair, such as PARPi. However, there are some pitfalls in this study. Although an independent dataset was used to validate the prognostic model and cell studies were performed to uncover the vital role of m6A-associated genes in metastatic PCa, other animal and clinical studies should be performed. Moreover, the present study is largely a bioinformatic analysis, and potential underlying mechanisms need to be further studied.

## Data Availability Statement

The original contributions presented in the study are included in the article/**Supplementary Material**. Further inquiries can be directed to the corresponding authors.

## Author Contributions

QL and JW designed the experiment. ZL performed some experiments. QL performed some experiments and finished the manuscript. LH performed some experiments. JW and HX participated in the experimental design and supervised the manuscript. The final version of the manuscript was read and approved by all authors.

## Funding

This work was funded by the National Natural Science Foundation of China (81771573).

## Conflict of Interest

The authors declare that the research was conducted in the absence of any commercial or financial relationships that could be construed as a potential conflict of interest.

## Publisher’s Note

All claims expressed in this article are solely those of the authors and do not necessarily represent those of their affiliated organizations, or those of the publisher, the editors and the reviewers. Any product that may be evaluated in this article, or claim that may be made by its manufacturer, is not guaranteed or endorsed by the publisher.

## Supplementary Material

Supplementary Figure 1 Comparison analysis of distinct m6Ageneclusters. **(A)** Unsupervised clustering of m6A phenotype-related genes in metastatic prostate cancer samples. Samples were separated into two different groups, termed m6Agenecluster.A and m6Agenecluster.B; **(B)** Kaplan–Meier curves show that there is a significant correlation between m6A-modified genomes and the overall survival rate; **(C)** The expression of 21 m6A regulator genes in two gene sets. The upper end, line and lower end of the box denote 1/4 quartiles, median and 3/4 quartiles, respectively; the black dots represent abnormal values; significant differences were determined by t test.

Supplementary Table S1 Sample information.

Supplementary Table S2 Copy number variations of 21 m6A regulators in primary, metastatic, adenocarcinoma and NEPC samples.

Supplementary Table S3 Cellular interactions.

Supplementary Table S4 m6A clusters.

Supplementary Table S5, 6 Gene Set Variant Analysis between m6A. Cluster A and m6A. Cluster B.

Supplementary Table S7, 8 The expression and mutation of specific genes between m6A. Cluster A and m6A. Cluster B.

Supplementary Table S9 The ARV7 score and ARscore between m6A.Cluster A and m6A.Cluster B.

Supplementary Table S10 Gene Set Variant Analysis between m6A. Cluster A and m6A. Cluster B based on the gene sets constructed by Mariathasan et al.

Supplementary Table S11 The expression levels of m6A regulators between m6A.Cluster A and m6A.Cluster B.

Supplementary Table S12 Metastatic prostate cancer-related differentially expressed genes (DEGs).

Supplementary Table S13 KEGG analysis for DEGs.

Supplementary Table S14 m6AGene clusters.

Supplementary Table S15 Category-related DEGs.

Supplementary Table S16, 17 m6Ascore.

Supplementary Table S18 Correlation analysis of m6Ascore and feature genes.

Supplementary Table S19 CNV numbers between m6Ascore high groups and low groups.

Supplementary Table S20 Survival analysis between m6Ascore high groups and low groups.
