# Supplementary figures and images for: Molecular Characterization and Clinical Relevance of N6-Methyladenosine Regulators in Metastatic Prostate Cancer
Source: Front Oncol. 2022 Jun 22;12:914692. doi: 10.3389/fonc.2022.914692 (PMC9257042; doi:10.3389/fonc.2022.914692)

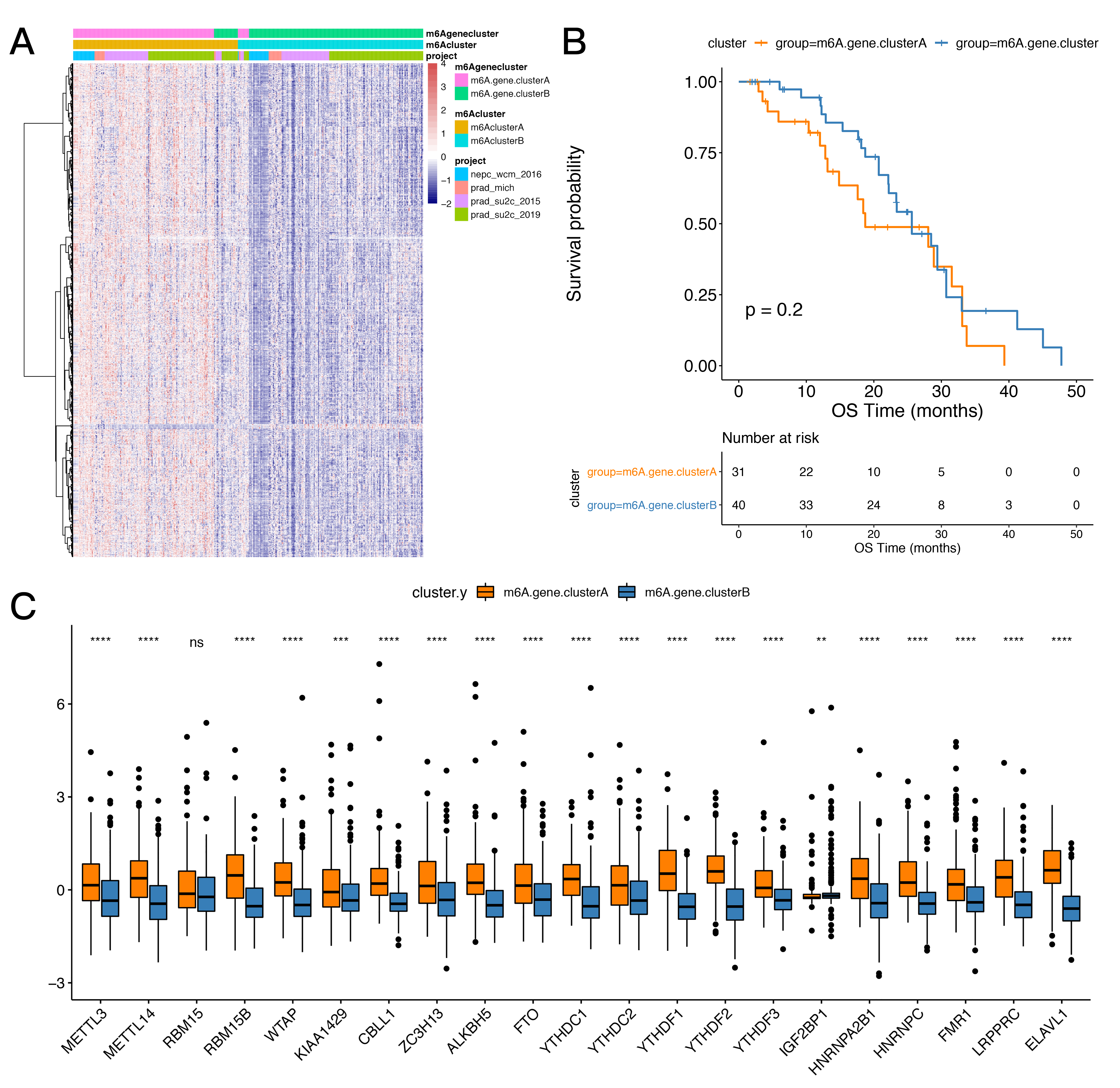

Supplement: Supplementary Figure 1 — Comparison analysis of distinct m6Ageneclusters. (A) Unsupervised clustering of m6A phenotype-related genes in metastatic prostate cancer samples. Samples were separated into two different groups, termed m6Agenecluster.A and m6Agenecluster.B; (B) Kaplan–Meier curves show that there is a significant correlation between m6A-modified genomes and the overall survival rate; (C) The expression of 21 m6A regulator genes in two gene sets. The upper end, line and lower end of the box denote 1/4 quartiles, median and 3/4 quartiles, respectively; the black dots represent abnormal values; significant differences were determined by t test. [file Image_1.tif]
